# Analysis of Maternal and Child Health Indicators in an Area at Paraná State, Brazil

**DOI:** 10.1155/2013/495178

**Published:** 2013-12-30

**Authors:** Emiliana Cristina Melo, Ana Beatriz Guedes Ribeiro, Rosana Rosseto de Oliveira, Robsmeire Calvo Melo Zurita, Thais Aidar de Freitas Mathias

**Affiliations:** ^1^Nursing Post-Graduate Program, Maringá State University, PR, Brazil; ^2^Northern Paraná State University, PR, Brazil; ^3^The State Department of Health (SESA), Municipal Health Department, Maringá, PR, Brazil

## Abstract

The aim of this study was to analyze maternal and child health indicators and infant mortality rate (IMR) at the cities located at the 18th Health Division (HD) in Parana State, Brazil. In this ecological study we analyzed all live births and infant deaths which occurred from 2000 to 2009 at the 18th HD, collecting data from the Mortality Information Database and the Live Births Information Database. The variables assessed were grouped into maternal, pregnancy and delivery, and neonatal variables. The analysis was conducted using the mean percentage of each variable and the IMR calculated for both periods: from 2000 to 2004 and from 2005 to 2009. The IMR was reduced considerably, following Brazil's and Paraná State's trend. Maternal indicators went down regarding the mean percentage of teenage mothers and low education, whereas they went up regarding mother with 35 years old or older and mothers without a partner. Pregnancy indicators showed increased prematurity and cesarean birth. Neonatal indicators raised in black/brown skin color and low birth weights percentages. This study provides a better understanding of maternal and child health in the cities located at the 18th HD, supplying grounds to plan actions regarding the real needs of each specific city.

## 1. Introduction

Maternal and child health is a priority in Brazil's agenda of health programs and actions, as well as in several countries. The concept of maternal and child health has expanded over the years, which also includes a set of knowledge, practices, and attitudes that aim at the promotion of healthy pregnancies, deliveries, and births and also the prevention of maternal and child mortality [1]. Brazil, as many other areas in the world, has been showing a reduction in infant mortality rate (IMR), mainly due to sanitary investments and increased health services accessibility [2].

In 1990, Brazil's infant mortality rate was 47.1 deaths by a thousand live births. In 2009, it went down to 22.5 by a thousand live births [3]. In Paraná State, infant mortality has also decreased in the last years. In 2001, this rate was 17.4 by a thousand live births, and in 2008, this rate declined to 12.1 by a thousand live births, showing a 30.4% reduction. However, important inequities are observed among the cities located at the 18th Health Division area, which must be highlighted and addressed by health managers while devising prevision and distribution of resources and services, as well as health professionals' qualification [4].

Studies conducted in Paraná State expose inequities in maternal, pregnancy, and neonatal health indicators among cities, showing a disadvantage in health and higher risk for infant mortality in some cities to detriment of others [4, 5]. Besides, a study that analyzed maternal and neonatal characteristics in host-cities of Health Divisions in Paraná State pointed to disadvantages at the 18th Health Division host-city compared to other host-cities. This may be influenced by the negative results presented by some cities under this Health Division management [4].

It is fundamental to acknowledge and understand the complex process of birth and factors that interfere with it, in order to deliver quality health assistance to mother and child. Furthermore, this can improve and rationalize health services in all stages of the reproductive cycle, giving priority to prevention, recuperation, and life maintenance actions. Also, this provides direction and taking preventive and curative measures in an adequate manner to suit the local scenario [6].

To reestablish the coherence between the health situation and the health services system, Family Health Strategy (FHS), it is necessary to involve the implantation and implementation of health services networks (HSNs). This is a new way to organize the health system into integrated networks which allow delivery of health care with effectiveness, efficiency, security, quality, and equity regarding health conditions for the population living in cities at Paraná State [7]. The HSNs model is taking place in Paraná State through a qualification process of health professionals included in this network, who are attending workshops regarding Primary Health Care (APSUS), more specifically in the maternal and child area with the implementation of the “Paraná's Mother” Program [8].

Considering this context, the aim of this study was to analyze maternal, pregnancy, and neonatal health indicators as well as the infant mortality rate in cities located at the 18th Health Division in Paraná State from 2000 to 2009. This will provide a profile of the situation in this area and will contribute to decision making towards preventive and assistance actions suitable to the local context.

## 2. Methods

This is a quantitative ecological study which analyzed all live births and infant deaths which occurred from 2000 to 2009 at the 18th Health Division (HD) in Paraná State.

The HDs are territorial areas identified to organize a network of health actions and services in order to ensure the application of the constitutional rights of accessibility, equity, and integral care. At the State of Paraná there are 22 HDs and the 18th is located in Northern Paraná State. It includes 21 cities and its host-city is Cornélio Procópio [9].

Data was collected from the Mortality Information Database (SIM) and the Live Births Information Database (SINASC) that were available online at the Information Technology Department website sponsored by the Single Health Unit (DATASUS). To analyze child health situation from cities located at the 18th HD, the IMR was calculated using the ratio between infant deaths (children younger than a year old) and live births during the same period and place multiplied by a thousand.

Maternal, pregnancy, and neonatal variables were grouped into categories. *Maternal variables* included age (<20 years old and >35 years old), education (<8 schooling years), and marital status (mothers without a partner, including singles, widows, separated, or divorced). *Pregnancy and delivery variables* included number of prenatal appointments (none and <7 appointments), duration of pregnancy in weeks (<37), and type of birth (cesarean). Finally, *neonatal variables* included Apgar scores (<8 in the 5th minute), birth weight (<2500 grams), and race (black and brown skin color).

Analysis was conducted using the mean percentages of each variable calculated for two periods, from 2000 to 2004 and 2005 to 2009, in each city. This allowed the comparison of indicators through the decade. Mean percentage was calculated by adding infant deaths of live births from each studied period. Missing data were not included in the analysis.

This research was approved by the Research Ethics Committee of the Nursing Undergraduate Course (CEP/CGE) from Northern Paraná State University, protocol no. 049/2011, under the direction of the National Research Council resolution no. 196/96.

## 3. Results and Discussion

According to data obtained from SINASC and SIM, from 2000 to 2009, there were 30,134,197 live births in Brazil, and 532,769 (1.7%) of those infants died earlier than completing one year old. In Paraná State, there were 1,600,390 live births from which 24,562 (1.5%) died. At the 18th HD in Paraná State, in the same period, 32,783 children were born corresponding to 2.0% of Paraná State births and the infant mortality accounted for 615 deaths in the same period, which corresponds to 1.8% of deaths compared to live births at this division. This rate is higher than the Brazilian one and the group of cities in Paraná State rates (data not presented here).

In the cities located at the 18th HD there was a decrease in IMR during the 10 years of study, accounting for 18.9 in the first period and 15.2 in the second period, following Brazil's trend of reduction (19.4 and 15.9) and the group of Paraná State cities (17.2 and 13.5) in the corresponding periods, as presented in Table 1.

Although there were disparities between neighboring cities, a considerable decrease in IMR at the 18th HD was identified. Some cities such as Andirá (dropped from 55.7 to 14.6), Santa Cecília do Pavão (dropped from 36.5 to 4.2), Santa Amélia (dropped from 25.4 to 5.7), and Abatiá (dropped from 30.5 to 11.7) presented high mean IMR from 2000 to 2004 but reached better results than Brazil and Paraná considering the period from 2005 to 2009 (Table 1).

On the other hand, we detected that cities with improved mean IMR in the first studied period, such as Santa Mariana (14.1), Cornélio Procópio (13.6), and Nova Santa Bárbara (6.4), conversely from what is expected, presented an increased IMR during the second period, going up to 21.3, 14.1, and 18.2, respectively (Table 1). This circumstance suggests the need for focused analysis and more attention to local public policies.

Infant mortality reduction may be due to several factors, including improvement of population's access to health services and resources, development of public policies towards maternal and child health, and also improvement of the region's developmental pattern and mother's higher education, and improvement of life conditions, income distribution, and environmental changes mainly regarding basic sanitation [10].

Despite the positive scenario, with reduced IMR in the 18th HD, Paraná, and Brazil, the IMR are still high, especially compared to developed countries such as Japan, Canada, Germany, Italy, and Spain, where rates range from three to six deaths by a thousand live births [11]. This leads to the thought that economic and social factors are still reflecting in the quality of life and health of the population from different geographical areas, even within the same country, demonstrating the heterogeneity between these territories [12].

Many researches relate infant mortality to socioeconomic conditions and to health care quality and access [2, 4]. It is worth mentioning the influence of socioeconomic characteristics in child's health conditions, regardless of age; hence low income and mother's education often determine precarious access to health services and technologies. Also, their knowledge about basic child needs is poor, which could be observed in the cities of Nova Fátima, Nova Santa Bárbara, Ribeirão do Pinhal, and Santa Mariana, that presented higher IMR means and mother's low education of more than 40% with less than eight schooling years (Table 2).

The analysis of maternal variables, which can be considered suitable indicators for risk or vulnerability conditions at birth, highlighted the reduction of percentage means regarding teenage and low education mothers. Conversely, percentage means of mothers older than 35 years old and without a partner rose during the studied period.

We observe a general reduction of teenage mothers, both in the cities located at the 18th HD (from 25.9% to 22.5%) as well as in Paraná State (from 21.6% to 20.2%) and in Brazil (from 22.7% to 20.9%). Some cities at the 18th HD presented lower percentage mean of teenage mothers compared to Paraná and Brazil during both periods, especially Cornélio Procópio (20.6% in the first period and 16.5% in the second period) and Rancho Alegre (18.6% to 17.2%). The opposite situation took place in the cities of Abatiá, São Jerônimo da Serra, and Uraí, where teenage mothers' rates increased during the studied period (Table 2). This represents increased risk for maternal and infant mortality at these cities as well as the existence of inappropriate educational public policies to prevent pregnancy in adolescence.

Children born to teenage mothers present higher chance of low birth weight and prematurity, which are substantial consequences to many morbidities and mortalities during childhood and can lead to future problems such as cognitive deficits, poor school performance, and behavioral issues [13].

In the same way as a result of this study, the percentage of teenage mothers have been decreasing around the world, mainly in developed and developing countries. This fact suggests the association between contraceptive techniques and its free distribution in basic health centers and also the existence of programs focused on sexual education for this age [14].

The mean corresponding to births by mothers older than 35 years old at 18th HD cities has progressively increased from 8.2% during the first period to 9.3% during the second period. In Paraná State, this rise was even higher, from 7.7% to 10.7%. In Brazil, it has increased from 8.9% to 9.7%. Individually, the 18th HD cities followed this trend, except for Santo Antônio do Paraíso (which dropped from 10.1% to 9.5%), Andirá (9.0% to 7.9%), Sapopema (7.2% to 6.1%), Congonhinhas (8.4% to 7.1%), and Sertaneja (7.8% to 5.4%), where the percentage of mothers older than 35 years old went down (Table 2).

The scientific literature identifies mothers older than 35 years old with risk for maternal and infant morbimortality and anomalies caused by chromosomal abnormalities that often happen to older women [15]. However, advanced maternal age has been associated with better socioeconomic status; hence these mothers may have more education which leads to improved work and wage conditions [4].

As for maternal formal education, this indicator helps show improvement in population's development and life condition. In the first period, 57.7% of mothers with children born at 18th HD cities presented less than eight schooling years, while in the second period, this percentage decreased to 42.5%. This trend was also observed in Paraná State (49.7% to 32.3%) and Brazil (54.3%, to 41.5%) during the same periods (Table 2).

The reduction of mean percentages regarding maternal low education occurred in all cities located at the 18th HD; the most important ones were noted at Abatiá (61.8% to 30.2%), Andirá (50.5% to 22.1%), and Ribeirão do Pinhal (77.7% to 42.6%). It draws attention to that the cities of Sapopema and São Jerônimo da Serra presented the highest rates and the lowest reduction of percentage means concerning maternal low education indicators comparing both periods. In Sapopema, it went from 78.7% in the first period to 70.2% in the second and from 71.9% to 60.1% in São Jerônimo da Serra. The cities of Abatiá, Andirá, Cornélio Procópio, and Itambaracá presented better results compared to Paraná State and Brazil (Table 2).

Maternal education is considered a sign for obstetric risk. Cities with higher percentage of pregnant women with more than eight schooling years have improved rates of prenatal followups. This happens because higher education may indicate better socioeconomic situation, consequently, leading to more access to health services, which in turn reduces infant morbimortality rates [16].

It is worth mentioning that cities that presented elevated IMR also presented high percentage of mothers with less than eight schooling years, as shown in Tables 1 and 2.

Concerning mothers' marital status, there was a considerable rise in number of mothers without a partner in Brazil, in Paraná State, and also in cities located at the 18th HD. During the first period, the 18th HD cities presented the mean percentage of 22.0%, which increased to 43.3% in the second period. Considering all cities in Paraná State, this rate was slightly higher, going up from 25.4% to 50.3%. In Brazil, the mean percentage was 42.3% in the first period and 62.2% in the second period (Table 2).

This rise in the percentage of women that take care of their children without the presence or assistance of their father has become more common and is considered a major change in family structure of several countries. This is also noted in Brazil, Paraná State, and cities at the 18th HD. This modification is part of a much broader process that involves socioeconomic and cultural changes, originated by a transformation in behavior patterns, such as increased types of relationship between genders, elevated number of separation and divorce, new sociability patterns and gender relations, and the fact that much more women are taking formal and informal jobs [17].

The absence of a partner during pregnancy and the child's first years may lead to a series of consequences to the mother, both physically and psychologically, and might lead to negative outcomes. Besides, mothers without a partner may present difficulties in strengthening the bond with the child. The affection offered by the partner may influence the development of maternal functions towards the baby, minimizing inability or weakness feelings regarding this new role, hence improving both the mother's and the child's wellbeing [18].

As far as pregnancy and delivery variables are concerned, they may be related to quality of health care assistance, which allows the verification of potential flaws in pregnancy health assistance. In the cities located at the 18th HD, the mean percentage of prematurity and cesarean deliveries increased, whereas the mean percentage of mothers who did not attend any or less than seven prenatal appointments decreased, comparing both studied periods.

The mean percentage of mothers who delivered their babies with less than 37 gestational weeks, in other words, premature babies, increased from 6.4% to 6.6%. This also occurred in Paraná State, rising from 6.5% to 6.7% and in Brazil, from 6.4% to 6.7%, during both periods (Table 3). Prematurity rates also increased in most studied cities, particularly in Nova Santa Bárbara (5.1% to 10.7%), followed by Ribeirão do Pinhal (3.9% to 7.8%), pointing out that these areas may have a higher risk for infant and, specially, neonatal mortality. The cities of Santa Mariana (11.9% to 8.2%), Santa Amélia (6.9% to 4.6%), and Sertaneja (7.0% to 5.1%) experienced the opposite situation, where mean percentages of premature deliveries decreased when compared between both periods (Table 3).

The percentages regarding cesarean deliveries displayed an increase during the analyzed periods. At the 18th HD, the mean percentage in the first period was 45.9% and 53.4% in the second period. In Paraná State the same percentage also went up from 45.9% to 53.4%, and in Brazil it raised from 39.2% to 46.6% (Table 3).

All analyzed cities had their cesarean percentages elevated, except for Congonhinhas, which decreased from 40.7% to 40.3%. Sapopema and Rancho Alegre presented the highest rise, from 24.8% to 46.2% and 48.8% to 70.1%, respectively. Sapopema was the city with the lowest percentage of cesarean deliveries during the first period. The following cities displayed elevated mean percentages during the last studied period: Santo Antônio do Paraíso (72.9%) and Rancho Alegre (70.1%) (Table 3).

The growth of cesarean births is not an isolated fact in the cities located at the 18th HD. This trend has been observed in all Brazilian territory during the last few years, and the numbers are even higher at private health services [19]. The reasons that explain this reality are not related to increased obstetric complications, which validate the medical indication for this procedure, but socioeconomic and cultural issues [19].

The surgical intervention to accomplish a delivery is strongly associated with a woman's education level, which increases progressively according to the number of schooling years. It is also more common between white women and those who attended several prenatal appointments, explaining the association with women with favorable socioeconomic situations [20].

In this study, the cities of Andirá, Cornélio Procópio, Leópolis, Santo Antônio do Paraíso, Sertaneja, and Uraí simultaneously presented increased mean percentages with improved results regarding mother's education, also displaying high mean percentages for cesarean births above 50%.

The factors that may interfere in the decision to have a cesarean delivery involve the obstetric organization, in which medical doctors are able to schedule the date of the intervention. Besides, mothers often feel hesitant about complications that could happen during vaginal labor. Another factor to be taken into account is that epidural anesthesia is not contemplated during vaginal births paid by the public health system. Furthermore, women's cultural values, fear of vaginal lesions that may change their anatomy, and belief that cesarean births are less risky than vaginal birth make them opt for cesarean deliveries [21].

The consequences related to this event include increased risk of maternal and perinatal morbidity and mortality, elevated costs and technological demands, increased occurrence of prematurity due to the inefficiency to precise the gestational age, and consequently low birth weight and Apgar scores. Additionally, there is a higher reliance on medical drugs and extended hospital stay, both for the mother and child, increasing the risk of being exposed to hospital infections [22].

As for prenatal appointments, the evolution was positive, because mean percentages of women who did not attend any appointments went down from 1.0% to 0.6% at the 18th HD. Paraná and Brazil also followed this trend, decreasing from 0.9% to 0.7% and 3.7% to 2.1%, respectively (Table 3).

Among the studied cities, only two presented the opposite situation, where percentages went up concerning the mentioned indicator, Andirá (0.2% to 0.3%) and Santa Amélia (0.7% to 1.0%). In some locations, records of mothers without prenatal appointments were not found for both periods such as Nova América da Colina and during the second period it happened in Santa Cecília do Pavão and Rancho Alegre (Table 3).

However, it is important to clarify that those cities which did not record any mother without prenatal appointments present low demographic density (<5000 population) [3]. At these areas, there was only one Basic Health Center or Family Health Team (FHT) to coverage most of the population. This feature contributes to early identification of women and consequently increases their access to health services during pregnancy; that is why FHT existence and adequacy are so important to Brazilian cities.

Mothers who attended two to six prenatal appointments during pregnancy accounted for the mean percentage of 36.4% from all pregnant women who lived in the 18th HD cities during the first period and 23.3% at the second period. This tendency was also observed in Paraná State, going down from 32.5% to 22.8%, and in Brazil decreasing from 45.4% to 41.0% mothers without suitable coverage of prenatal appointments (Table 3).

Out of all 18th HD cities, just Bandeirantes and Itambaracá presented the contrary situation, where the mean percentage of mothers without the minimum of seven prenatal appointments went up from 25.6% to 30.0% and 24.8% to 28.9%, comparing both periods. The following cities managed to reduce the mean percentage of mothers who could not attend any appointment: São Sebastião da Amoreira (60.7% to 31.0%), Sapopema (68.5% to 41.8%), Uraí (37.2% to 14.4%), and Santa Mariana (45.3% to 23.4%). At Ribeirão do Pinhal, although presenting a decrease in both periods, higher mean percentages of mothers with inadequate prenatal care were observed (from 69.3 to 61.9%), suggesting further analysis which focused on the city to identify what leads to it.

Attending prenatal appointments minimizes the risks for maternal and infant morbimortality; hence quality assistance to women during pregnancy is fundamental to prevent morbidities and risk factors that interfere with mother's and child's health. Besides, it provides adequate assessment to indicate cesarean births accurately [1].

Regarding neonatal variables, which may be influenced by socioeconomic characteristics along with quality assistance to pregnancy and delivery, we observe proportional elevation in black skin and low birth weight in 18th HD cities, Paraná State, and Brazil. Apgar scores were disappointing at 5th minute, showing slight reduction, comparing the first and second periods.

Considering the race variable, mean percentages of black increased at 18th HD cities compared to Paraná State, going up from 9.6% during the first period to 12.3% at the second period. In Paraná State, it was increased from 5.5% to 6.5% and in Brazil from 40.2% to 47.7% (Table 4).

Among analyzed cities, some presented diminished mean percentages regarding black race, such as Santa Amélia (23.4% to 14.9%), Bandeirantes (12.3% to 7.4%), Itambaracá (11.0% to 6.9%), Rancho Alegre (12.1% to 9.1%), and Santa Mariana (15.5% to 12.6%). Moreover, in other cities there was a rise in mean percentages from the first to the second period, for example, Sapopema (6.8% to 23.5%), Santa Cecília do Pavão (8.0% to 22.8%), São Sebastião da Amoreira (14.7% to 23.7%), and Nova Santa Bárbara (10.2% to 19.0%) (Table 4).

The expansion of black population in Brazil has also been observed by the demographic census conducted by IBGE in 2010, when nearly 97 million Brazilian declared to be black and brown, and 91 million people declared to be white. This was the first time that black/brown race was considered the majority of the national population [3].

This inversion may be caused by the elevated fecundity rate of black women compared to white ones, and the potential increased number of people who considered themselves black/brown skinned [10]. Moreover, this may be due to improved access to health assistance during pregnancy experienced by black women, which in turn reduces the risks for fetal mortality.

It is also important to note that, although there have been some changes in racial concepts and prejudice, being a black-skinned person in Brazil still represents great social and economic inequalities. This reflects the higher vulnerability this population is exposed to, especially children and newborns [11]. This information must be carefully analyzed by health assistance administrators and managers, in order to make decisions that imply improved access and quality of health care to black/brown pregnant women and newborns.

As for low birth weight, we observed that 7.7% of children born at the 18th HD during the first period and 8.1% born in the second period had less than 2500 grams at birth. This rise was also present in Paraná State (8.3% to 8.4%) and Brazil (8.0% to 8.2%) at the same periods (Table 4).

In general, cities located at the 18th HD followed this trend, mainly Nova Santa Bárbara (8.9% to 15.4%), Leópolis (6.1% to 10.1%), Nova América da Colina (6.1% to 9.7%), and Nova Fátima (6.1% to 9.2%) presenting more elevated mean percentages in the first period compared to the second period. Conversely, other cities had their mean percentages decreased considering low birth weight, such as Sertaneja (9.7% to 4.3%) and Santo Antônio do Paraíso (9.0% to 6.6%) (Table 4).

Worldwide, low birth weight is considered one of the major problems in infant public health. It is often related to prematurity and low prenatal coverage. Among its main consequences are the elevated rates of infant mortality and, in some cases, they can lead to impairment in neurologic and intellectual health during infancy [23].

The rise in mean percentages of babies born with low weight observed in this study endorses other studies [4, 24]. This situation may be associated with increased prematurity and cesarean birth in all studied cities, substantially in Cornélio Procópio, Leópolis, Nova Fátima, Nova santa Bárbara, and Santa Mariana. These cities simultaneously showed high mean percentages for prematurity and cesarean birth and low birth weight.

Disappointing Apgar scores (less than 8 at the 5th minute) were detected in 2.0% of births which occurred at 18th HD cities during the first period and in 1.5% at the second period. This reduction was also observed in Paraná State (2.3% to 1.9%) and Brazil (3.0% to 2.2%) (Table 4).

Mean percentages of this indicator also decreased in most studied cities, notably in Rancho Alegre (4.2% to 1.5%) and São Sebastião da Amoreira (3.9% to 2.2%). Among the cities with increased disappointing Apgar scores, the most significant ones were Nova Santa Bárbara (2.8% to 3.9%) and Itambaracá (1.1% to 2.2%) (Table 4).

The Apgar score is an assessment tool used immediately after birth; it measures the newborn's vitality. When scores are below 8, the baby has asphyxiation symptoms. The lowest the score, the worst the baby's condition, indicating risk for neonatal morbimortality. When this happens, there is the need for reanimation and intensive care which may lead to neonatal death in 70% of cases [25].

As such, although it is very slight, the decreased proportion of live births with Apgar scores ranging from zero to seven at the 5th minute established in this research might be considered as a positive aspect regarding the improvement in maternal and infant health.

## 4. Conclusion

The aim of this study was to analyze indicators regarding maternal, pregnancy, and neonatal health as well as the IMR in cities located at the 18th HD in Paraná State, from 2000 to 2009. During this ten-year period, we observed changes in indicators development with positive advances in maternal and child health at the 18th HD in Paraná and also overall in Paraná and Brazil. This was possibly due to the improvement in health services accessibility and changes in socioeconomic and cultural profiles of this population.

Health indicators related to the mother, pregnancy, and newborn displayed satisfying results, whereas there was a reduction in percentage of teenage mothers and low education and a rise in numbers of prenatal appointments. On the other hand, situations that may increase the risk for maternal and child morbimortality were identified, such as increased number of mothers without a partner, cesarean births, births earlier than 37 weeks, and low birth weight.

Improvements in maternal and child health were established between the populations of the 18th HD; yet, some percentages remain dissatisfying, mostly regarding cesarean birth, that may contribute to increased prematurity and low birth weight.

The results of this investigation provide a better understanding of maternal and child health in cities located at the 18th HD in Paraná State. They serve as grounds to plan health actions, especially in Bandeirantes, Nova Fátima, Nova Santa Bárbara, Ribeirão do Pinhal, and Santa Mariana where elevated IMR are present when compared to other 18th HD cities, in Paraná State and Brazil.

Besides, some cities such as São Jerônimo da Serra, Ribeirão do Pinhal, Sapopema, Santa Cruz do Pinhal, and Santo Antonio da Amoreira among others deserve greater investments through public policies regarding education and health, because in these cities some indicators such as percentage of teenage mothers, and low education, low number of prenatal appointments remain constantly elevated.

The 18th HD should consider the real needs of each city under its responsibility, making specific decisions towards this unique population, essentially focusing on maternal and child population.

When population and health managers and professionals work together, it is possible to implement guidelines and actions that target health education and promotion. Substantially, nurses are fundamental to maternal and child health promotion due to their close relation with the population assisted by the Family Health Team and at Basic Health Centers.

The Brazilian Unified Health System (SUS) recently created a health policy named APSUS to permanently capacitate all health workers. It is currently in course at Paraná State and may lead to improvement in indicators through the implantation and implementation of stratified maternal and neonatal risks in all HD of the state.

Variables available at the SUS database, for example, SIM and SINASC, should be analyzed at central management level as well as by the health team in each city or Basic Health Unit. Although there are some limitations on using secondary data, decentralized data provide analysis in local level and allow the identification and frequent monitoring of this population's health status, highlighting differences between cities and coverage areas. Also, this information shows results and reflects health assistance delivered in that area, bringing up potential inequities about quality of life, education, and maternal and child health assistance.

Further studies should be done in order to better understand the health status of population living at 18th HD cities, especially those who show unsatisfactory mother and child health indicators. Conditions of institutions such as accessibility, quality, and effectiveness of health care assistance and effectiveness of health networks when referring to other services are some of issues that should be investigated.

## Figures and Tables

**Table 1:** Distribution of IMR means from the cities located at the 18th HD in Paraná State from 2000 to 2004 and 2005 to 2009.

City	1st period (2000 to 2004)	2nd period (2005 to 2009)
Abatiá	30.5	11.7
Andirá	55.7	14.6
Bandeirantes	20.3	18.4
Congonhinhas	20.3	13.4
C. Procópio	13.6	14.1
Itambaracá	25.5	13.4
Leópolis	20.9	15.9
Nova A. da Colina	21.1	12.5
Nova Fátima	22.4	22.0
Nova S. Bárbara	6.4	18.2
Rancho Alegre	21.7	5.4
Ribeirão do Pinhal	27.0	22.1
Santa Amélia	25.4	5.7
Santa C. do Pavão	36.5	4.2
Santa Mariana	14.1	21.3
Santo A. Paraíso	22.2	5.7
São J. da Serra	22.9	16.5
São S. da Amoreira	18.7	11.3
Sapopema	28.0	14.8
Sertaneja	16.5	15.8
Uraí	14.4	10.0
Total HD	**18.9**	**15.2**
Total Paraná	**17.2**	**13.5**
Total Brazil	**19.4**	**15.9**

Source: Brazilian Ministry of Health—DATASUS.

**Table 2:** Distribution of percentage means according to maternal variables, 18th HD, Paraná, and Brazil, from 2000 to 2004 and 2005 to 2009.

City	Age				Scholarity		Marital status	
	<20 years old		>35 years old		<8 schooling years		Without a partner	
	2000–2004	2005–2009	2000–2004	2005–2009	2000–2004	2005–2009	2000–2004	2005–2009
Abatiá	26.1	28.0	9.0	7.9	61.8	30.2	17.8	60.3
Andirá	26.1	23.5	5.3	7.0	50.5	22.1	20.4	21.0
Bandeirantes	23.2	19.8	8.5	10.0	55.8	33.5	16.0	48.4
Congonhinhas	28.6	25.6	8.4	7.1	62.9	31.8	14.1	46.0
C. Procópio	20.6	16.5	9.4	11.4	45.5	30.6	21.7	39.9
Itambaracá	29.8	22.8	4.0	6.8	62.0	48.6	19.8	55.8
Leópolis	24.5	22.5	7.6	8.7	51.9	37.5	23.1	45.5
Nova A. Colina	23.0	19.5	8.8	12.2	57.6	41.8	18.7	37.0
Nova Fátima	25.3	23.8	6.8	9.2	64.5	45.5	28.3	45.8
Nova S. Bárbara	26.4	25.9	8.4	9.6	50.7	42.4	38.8	47.6
Rancho Alegre	18.6	17.2	9.2	9.3	58.6	51.5	22.9	42.5
Ribeirão Pinhal	26.8	25.3	10.2	10.4	77.7	42.6	20.1	56.8
Santa Amélia	28.5	26.0	8.1	9.0	65.9	50.0	16.2	42.6
Santa C. Pavão	28.0	24.8	6.3	8.5	46.2	33.8	30.9	38.7
Santa Mariana	28.9	24.8	5.9	7.7	60.2	45.9	17.9	46.0
Santo A. Paraíso	22.5	21.7	10.1	9.5	48.6	34.3	16.4	31.7
São J. da Serra	28.5	30.5	9.1	8.3	71.9	60.1	17.8	42.5
São S. Amoreira	27.9	23.2	6.3	9.2	55.6	40.0	43.7	53.7
Sapopema	25.7	23.6	7.2	6.1	78.7	70.2	34.1	34.1
Sertaneja	27.3	21.9	7.8	5.4	58.4	39.0	17.7	41.6
Uraí	23.2	23.6	8.2	9.1	51.0	37.1	23.2	44.3
Total HD	**25.9**	**22.5**	**8.2**	**9.2**	**57.7**	**42.5**	**22.0**	**43.2**
Total Paraná	**21.6**	**20.2**	**7.7**	**10.7**	**49.7**	**32.3**	**25.4**	**50.3**
Total Brazil	**22.7**	**20.9**	**8.9**	**9.7**	**54.3**	**41.5**	**42.3**	**62.2**

Source: Brazilian Ministry of Health—DATASUS.

**Table 3:** Distribution of percentage means according to pregnancy variables, 18th HD, Paraná, from 2000 to 2004 and 2005 to 2009.

City	Prenatal appointments				Duration of pregnancy		Type of birth	
	None		≥7		<37 weeks		Caesarean	
	2000–2004	2005–2009	2000–2004	2005–2009	2000–2004	2005–2009	2000–2004	2005–2009
Abatiá	1.5	0.2	28.4	14.2	3.3	4.7	51.3	53.0
Andirá	0.2	0.3	19.2	4.8	3.0	4.4	49.8	57.7
Bandeirantes	1.5	0.5	25.6	30.0	9.5	6.4	44.7	50.3
Congonhinhas	0.5	0.8	26.3	19.9	3.7	3.4	40.7	40.3
C. Procópio	1.0	0.6	25.7	13.9	6.8	8.4	58.6	68.4
Itambaracá	0.8	0.4	24.8	28.9	7.9	8.8	45.0	54.8
Leópolis	0.7	0.5	28.9	14.8	6.5	6.7	52.7	58.4
Nova A. Colina	1.8	0.0	41.5	19.9	6.1	5.1	51.2	60.7
Nova Fátima	0.9	0.7	42.3	22.1	6.3	8.1	42.1	55.7
Nova S. Bárbara	0.6	0.4	45.7	34.2	5.1	10.7	43.0	54.0
Rancho Alegre	0.3	0.0	38.2	17.2	4.3	5.3	48.8	70.1
Ribeirão Pinhal	0.8	0.6	69.3	61.9	3.9	7.8	34.5	43.7
Santa Amélia	0.7	1.0	28.0	22.6	6.9	4.6	33.0	47.9
Santa C. Pavão	2.7	0.0	33.2	31.0	6.6	6.6	47.8	63.5
Santa Mariana	1.1	0.6	45.3	23.4	11.9	8.2	43.8	60.0
Santo A. Paraíso	0.0	0.0	32.0	11.1	5.3	7.3	61.2	72.9
São J. da Serra	2.1	1.3	57.8	38.4	3.7	5.2	31.9	44.3
São S. Amoreira	0.4	0.4	60,7	31.7	7.9	8.0	37.7	56.4
Sapopema	1.5	1.4	68.5	41.8	6.4	5.4	24.8	46.2
Sertaneja	0.7	0.6	30.3	13.2	7.0	5.1	47.0	59.3
Uraí	1.6	0.4	37.2	14.4	6.1	5.3	47.9	57.1
Total HD	**1.0**	**0.6**	**36.4**	**23.3**	**6.4**	**6.6**	**45.9**	**55.6**
Total Paraná	**0.9**	**0.7**	**32.5**	**22.8**	**6.5**	**6.7**	**45.9**	**53.4**
Total Brazil	**3.7**	**2.1**	**45.4**	**41.0**	**6.4**	**6.7**	**39.2**	**46.6**

Source: Brazilian Ministry of Health—DATASUS.

**Table 4:** Distribution of percentage means according to neonatal variables, 18th HD, Paraná, from 2000 to 2004 and from 2005 to 2009.

City	Race		Birth weight		Apgar score	
	Black/brown skin color		<2500 grams		<8 at 5th minute	
	2000–2004	2005–2009	2000–2004	2005–2009	2000–2004	2005–2009
Abatiá	16.6	20.1	7.7	6.6	1.3	1.5
Andirá	1.1	7.8	6.7	7.0	2.1	2.0
Bandeirantes	12.3	7.4	8.0	8.1	1.7	1.0
Congonhinhas	14.3	15.7	7.9	8.0	1.7	1.9
C. Procópio	7.7	10.6	7.9	8.7	1.6	0.8
Itambaracá	11.0	6.9	8.5	7.4	1.1	2.2
Leópolis	8.2	8.6	6.1	10.5	2.0	1.3
Nova A. Colina	4.5	11.3	6.1	9.7	1.8	2.2
Nova Fátima	7.9	11.3	6.1	9.2	1.6	1.5
Nova S. Bárbara	10.2	19.0	8.9	15.4	2.8	3.9
Rancho Alegre	12.1	9.1	5.9	8.8	4.2	1.5
Ribeirão Pinhal	5.9	7.8	6.2	9.1	3.3	2.5
Santa Amélia	23.4	14.9	9.5	6.7	1.8	1.8
Santa C. Pavão	8.0	22.8	10.7	7.5	2.8	1.7
Santa Mariana	15.5	12.6	8.9	9.2	1.8	1.7
Santo A. Paraíso	3.4	9.5	9.0	6.6	1.5	0.6
São J. da Serra	7.4	15.5	6.2	7.7	2.3	1.3
São S. Amoreira	14.7	23.7	9.0	7.9	3.9	2.2
Sapopema	6.8	23.5	9.0	8.0	2.6	1.1
Sertaneja	4.9	7.6	9.7	4.3	1.2	1.5
Uraí	17.2	22.8	7.8	6.3	1.3	1.0
Total HD	**9.6**	**12.3**	**7.7**	**8.1**	**2.0**	**1.5**
Total Paraná	**5.5**	**6.5**	**8.3**	**8.4**	**2.3**	**1.9**
Total Brazil	**40.2**	**47.7**	**8.0**	**8.2**	**3.0**	**2.2**

Source: Brazilian Ministry of Health—DATASUS.
